# Effects of dihydrotestosterone on osteoblast activity in curdlan-administered SKG mice and osteoprogenitor cells in patients with ankylosing spondylitis

**DOI:** 10.1186/s13075-020-02217-9

**Published:** 2020-05-24

**Authors:** Sungsin Jo, Eun-Ju Lee, Bora Nam, Juyeon Kang, Seunghun Lee, Jeehee Youn, Ye-Soo Park, Yong-Gil Kim, Tae-Hwan Kim

**Affiliations:** 1 Hanyang University Institute for Rheumatology Research, Seoul, Republic of Korea; 2 Division of Rheumatology, Department of Medicine, University of Ulsan College of Medicine, Asan Medical Center, Seoul, Republic of Korea; 3 Department of Rheumatology, Hanyang University Hospital for Rheumatic Diseases, Seoul, Republic of Korea; 4 Department of Radiology, Hanyang University Hospital, Seoul, Republic of Korea; 5 Department of Anatomy and Cell Biology, College of Medicine, Hanyang University, Seoul, Republic of Korea; 6 Department of Orthopedic Surgery, Hanyang University Guri Hospital, Guri, Republic of Korea

**Keywords:** Ankylosing spondylitis, Spinal ankylosis, Dutasteride, Curdlan-administered SKG mice, Dihydrotestosterone, Osteoprogenitors, Osteoblastic activity

## Abstract

**Background:**

Ankylosing spondylitis (AS) is characteristically male-predominant, and progressive spinal ankylosis affects male patients more severely; however, the hormonal effects in males with AS are poorly understood.

**Methods:**

In the present study, the regulatory effects of dutasteride, a 5-α reductase inhibitor that blocks the conversion of testosterone to dihydrotestosterone (DHT), were examined in curdlan-administered male SKG mice to determine spinal bone formation, bone metabolism-related markers, and interleukin (IL)-17A cytokine and T cell populations. In addition, the effects of DHT on primary osteoprogenitors from the facet joints of AS patients were assessed based on osteoblast-related parameters. DHT level was measured, and the correlation with modified Stoke Ankylosing Spondylitis Spinal Score (mSASSS) was analyzed in AS patients.

**Results:**

In curdlan-administered SKG mice, dutasteride treatment resulted in an increased accumulation of hydroxyapatite in the spine which was positively correlated with serum IL-17A levels. In the analysis of bone metabolism-related molecules, a decrease in sclerostin levels was observed in the sera in the dutasteride group. Continuous exposure to DHT resulted in fewer calcium deposits in AS osteoprogenitors during osteoblast differentiation. DHT-treated AS osteoprogenitors showed decreased osteocalcin and increased *DKK1* and *SOST1* mRNA expression, supporting the results of the in vivo experiments. Treatment with dutasteride upregulated bone formation in the spine of curdlan-administered SKG mice and DHT treatment downregulated osteoblast differentiation in vitro.

**Conclusions:**

Treatment with dutasteride affected the bone formation in the spine of curdlan-treated SKG mice, and DHT treatment attenuated osteoblast differentiation in vitro. Therefore, contrary to what could be expected if osteoblasts contributed to spinal ankylosis, DHT inhibition might increase rather than decrease the progression of spinal ankylosis despite the higher levels of DHT observed in many AS patients.

## Background

Ankylosing spondylitis (AS) is a male-predominant disease, and its symptomatic burden as well as the progression of spinal changes is more severe in male patients [1, 2]. Male hormone analysis in AS patients has been reported [3–5]; however, an obvious difference in the effects of hormones was not observed. The putative effects of male hormones are difficult to ascertain because AS is a multifactorial disease. Syndesmophytes are the first indicator of spinal ankylosis in AS. The syndesmophyte formation is orchestrated by a variety of immune cells in response to multifaceted factors including genetic factors such as HLA-B27, chronic inflammation, and environmental factors with mechanical stress [6–8]. Furthermore, interleukin (IL)-17A levels and the number of IL-17A-secreting cells are higher in male AS patients [9]. Exogenous IL-17A treatment of primary bone-driven cells in AS patients promotes osteoblast activity and differentiation [10]; however, the association between male hormones and pathogenic subset of immune cells or syndesmophyte formation remains unknown.

SKG mice possess a mutation in the ZAP70 gene, a key signaling molecule in T cells [11]. Although SKG mice have been used as an animal model of rheumatoid arthritis (RA), curdlan-administered SKG mice showed spontaneous inflammatory arthritis and developed spinal bony fusion over time [12–14]. Therefore, the curdlan-administered SKG mice have been suggested as a model of human spondyloarthritis.

Testosterone affects immune responses such as differentiation and cytokine production of T cells, B cells, and macrophages. Lower serum testosterone levels are associated with the future development of RA, especially in patients who are rheumatoid factor-negative [15]. In SKG mouse models, testosterone inhibits the development of arthritis, indicating an inhibitory role of testosterone in the development of RA in male patients [16]. However, serum testosterone levels in AS patients are significantly higher than those in RA patients but not different from the healthy population [17]. Therefore, considering the male predominance of AS and the non-reduced testosterone levels in AS patients, testosterone might be more involved in the pathogenesis of AS compared with other types of arthritis.

Dutasteride, a 5-α reductase (SRD5A) inhibitor that blocks the conversion of testosterone to dihydrotestosterone (DHT), has been clinically used for the treatment of aggressive prostate cancer and alopecia [18, 19]. Although dutasteride has been proven safe, the effects of decreased DHT levels in male AS patients undergoing long-term dutasteride treatment are unknown. In the present study, the effects of dutasteride on spinal ankylosis were examined in a spondyloarthritis animal model. Furthermore, the role of DHT in the differentiation of human osteoblasts was evaluated using primary AS osteoprogenitors.

## Materials and methods

### In vivo AS model

SKG mice were obtained from Dr. Sakaguchi (University of Osaka, Japan), and arthritis was induced as previously described [11–13]. Eight-week-old male SKG mice were intraperitoneally injected with 3 mg of curdlan (*n* = 20) or PBS only as a control (*n* = 6) at 0 and 2 weeks. Dutasteride (SML1221, Sigma, USA), a 5-α reductase inhibitor that blocks the conversion of testosterone to DHT, was used for in vivo experiments. An experimental diet containing dutasteride (41.2 mg/kg/day) was started at 9 weeks after the first curdlan injection (dutasteride group, *n* = 10) in half of the mice, and a normal diet was maintained in the remaining mice (curdlan group, *n* = 10). The clinical scores for peripheral arthritis were evaluated weekly. At 17 weeks after the first curdlan injection, whole-body imaging was performed using the fluorescent in vivo bisphosphonate agent OsteoSense® 680 EX (Mediso Ltd., Hungary). The mice were then sacrificed, and sera and splenocytes were simultaneously collected. The levels of bone metabolism-related molecules including receptor activator of nuclear factor kappa-Β ligand (RANKL), osteoprotegerin (OPG), dickkopf-related protein 1 (DKK1), and sclerostin (SOST) were analyzed using a Luminex multiplex assay, and IL-17A was measured using single molecule arrays. IL-17A secretory cells in the spleen were measured using flow cytometry. All mice were handled in accordance with the guidelines for animal care approved by the Animal Experimentation Committee of the Asan Institute for Life Science (2015-14-135).

### In vitro studies and sera analysis

Studies involving human materials were performed in compliance with the Helsinki Declaration and approved by the Ethics Committee of Hanyang University Hospital; written informed consent was obtained from all subjects (IRB-2014-05-002). Human bone tissues were obtained during surgery from the facet joints of 12 patients who fulfilled the 1984 modified New York classification criteria for AS. Primary AS osteoprogenitor cells were cultured as previously reported [10, 20, 21]. The effects of DHT (D-073, Sigma, USA) on primary AS osteoprogenitors were assessed by alkaline phosphatase (ALP) activity and staining, alizarin red staining (ARS) for calcium deposits, and qRT-PCR for osteoblasts-related genes [22]. RNA extraction (TRIzol, Thermo Fisher Scientific, USA) and cDNA synthesis (ReversAid, Thermo Fisher Scientific, USA) were performed following the standard protocols.

The qRT-PCR primers used were as follows: 18s RNA forward, 5′-GTAACCCGTTGAACCCCATTC-3′; 18s RNA reverse, 5′-CCATCCAATCGGTAGTAGCG-3′; *ALP* forward, 5′-ACGAGCTGAACAGGAACAACGT-3′; *ALP *reverse, 5′-CACCAGCAAGAAGAAGCCTTTG-3′; *OCN* forward, 5′-ATGAGAGCCCTCACACTCCT-3′; *OCN* reverse, 5′-CTTGGACACAAAGGCTGCAC-3′; *DKK1* forward, 5′-GGGTCTTTGTCGCGATGGTA-3′; *DKK1* reverse, 5′- CTGGTACTTATTCCCGCCCG-3′; *SOST1* forward, 5′-TGGCAGGCGTTCAAGAATGA-3′; *SOST1* reverse, 5′-GCCCGGTTCATGGTCTTGTT-3′.

Sera were collected from male subjects: 28 healthy donors (HC), 189 with AS, and 23 with RA. The DHT levels in the sera were analyzed using ELISA (KA1886, Abnova, Taiwan). Spinal radiographs of the AS patients were scored based on the modified Stoke Ankylosing Spondylitis Spinal Score (mSASSS) (Lee S).

### Statistical analysis

Statistical analyses were performed using GraphPad Prism software, version 6.0. The Mann-Whitney *U* test was performed for two-group comparisons, and *P* values < 0.05 were considered statistically significant. All results are presented as the mean ± standard error of the mean (SEM). Statistical Package for Social Science (SPSS) software was used for statistical analysis. Spearman’s correlation was used to determine the correlation between DHT and mSASSS.

## Results

**Fig. 1:**
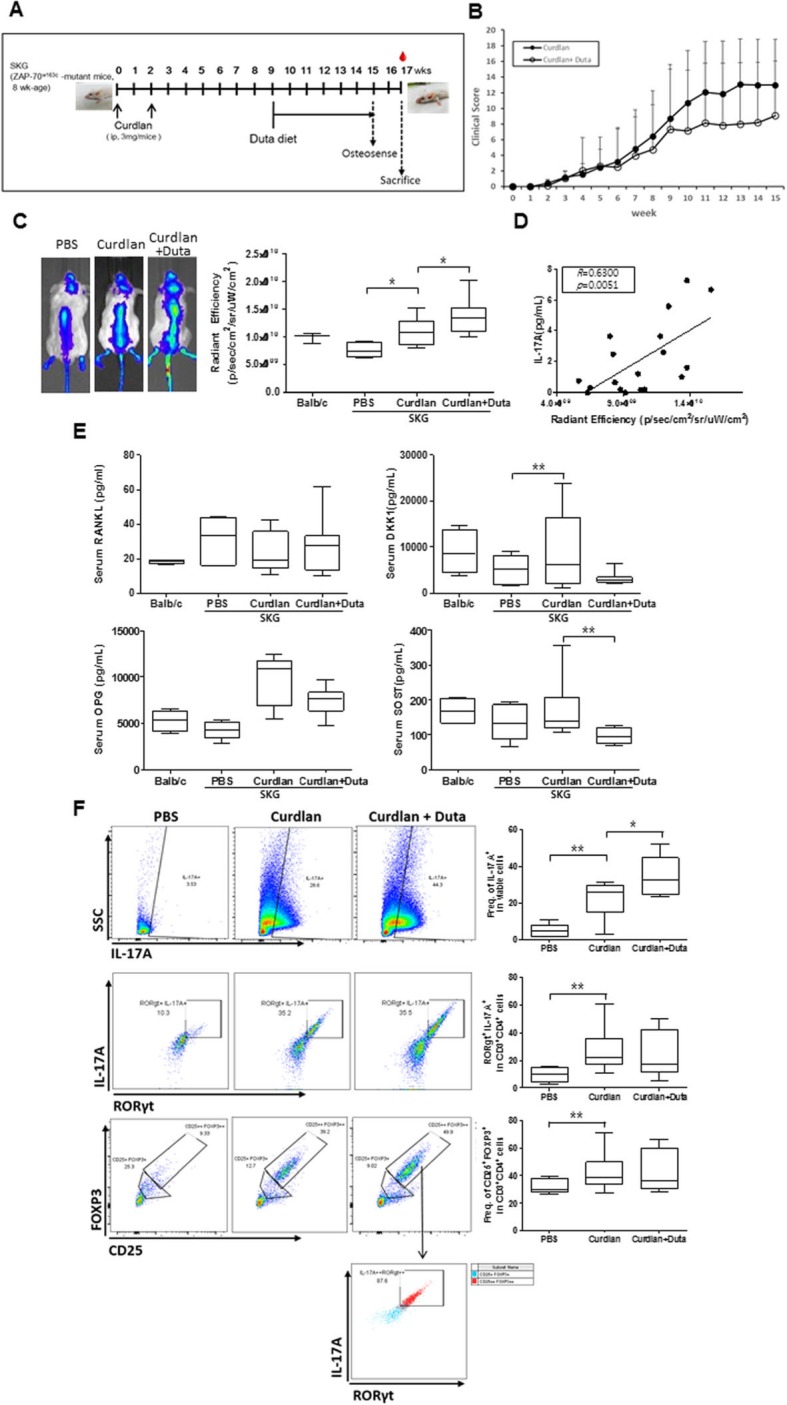
Effects of dutasteride on curdlan-administered SKG mouse model. **a** Experimental design. **b** Clinical arthritis scoring. **c** In vivo imaging after injection of OsteoSense® 680 EX probe and quantitative analysis of fluorescence values. **d** Correlation between serum IL-17A and bone mineralization. **e** Serum levels of bone metabolism-related molecules. **f** Flow cytometry plots showing the proportion of IL-17A^+^ cells, IL-17^+^RORγT cells (T_H_17), and CD25^+^FoxP3^+^ cells (Treg) among splenocytes. *p < 0.05, **p < 0.01

Curdlan-administered SKG mice were examined to determine the effects of dutasteride on the induction of arthritis and spinal progression in AS. The experimental design is shown in Fig. 1a. After starting the dutasteride diet, the clinical arthritis scores were not different between the dutasteride and curdlan groups (Fig. 1b). At 2 weeks before sacrifice, the accumulation of hydroxyapatite in the spinal region, which reflects osteoblast activity, was significantly increased in the dutasteride group compared with the curdlan group (Fig. 1c). The osteoblast activity was positively correlated with the IL-17A serum level (Fig. 1d). In the analysis of bone metabolism-related molecules, the OPG levels were increased in the curdlan group compared with PBS-treated SKG mice but were not different between the curdlan and dutasteride groups. However, the SOST levels were markedly decreased in the dutasteride group compared with the curdlan group (Fig. 1e). Among splenocytes, the population of IL-17A secretory cells was increased in all curdlan-administered mice, with larger increases in the dutasteride group compared with the curdlan group. However, the amount of T_H_17 cells and IL-17A^+^Treg cells were not significantly different between the dutasteride and curdlan groups (Fig. 1f). Collectively, these results indicate that treatment with dutasteride does not attenuate arthritis but does increase mineralization of the spine in curdlan-administered SKG mice, likely via the IL-17A pathway.

**Fig. 2:**
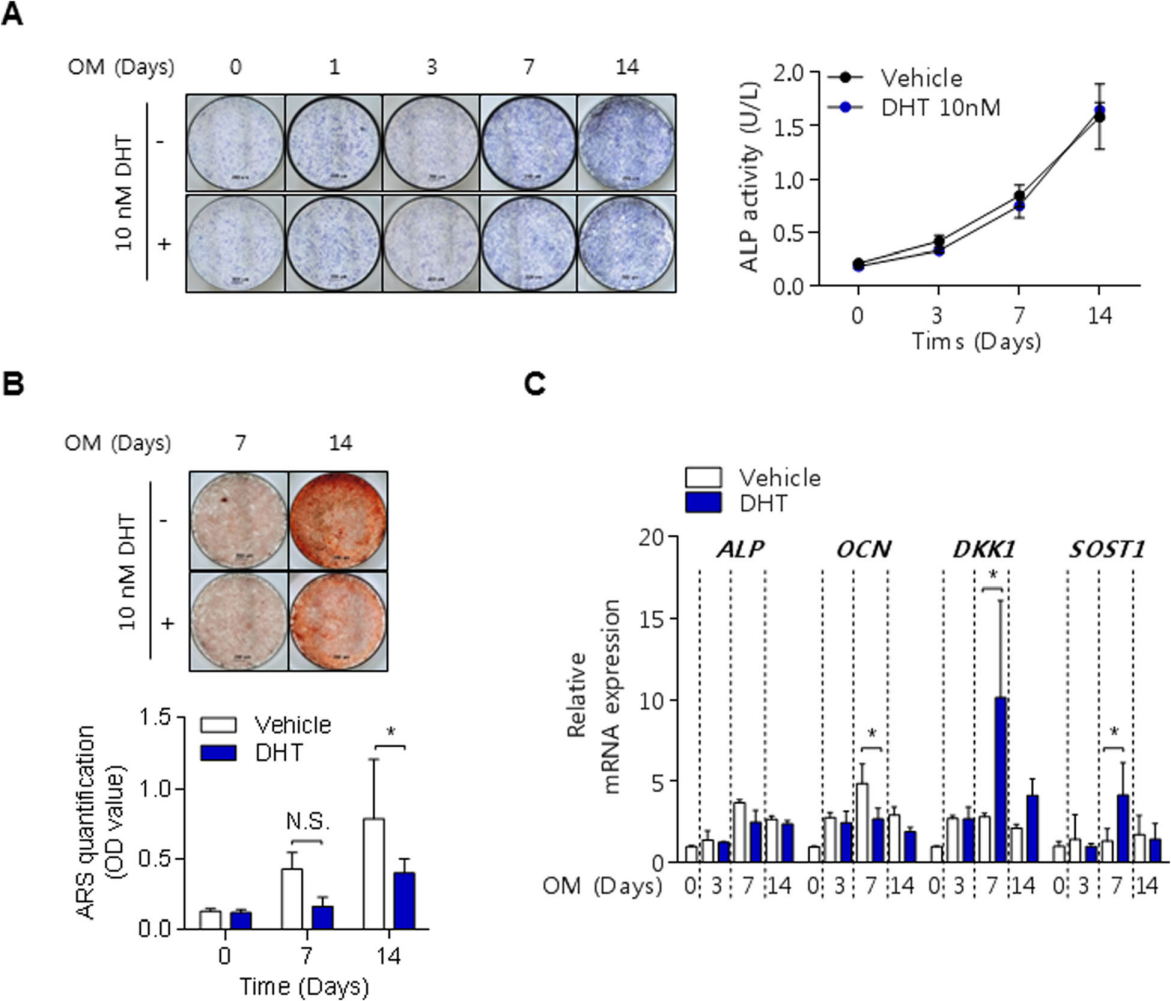
Effects of DHT on primary osteoprogenitor cells in AS patients. **a** ALP staining (left) and activity (right) during 14 days of culture in osteogenic media. **b** ARS (upper) and quantification (lower) during 14 days of culture in osteogenic media. **c** qPCR of osteoblast-related gene expressions. *p < 0.05

Based on the in vivo results with dutasteride treatment as a DHT inhibitor, the effects of DHT on AS osteoprogenitor cells during osteogenic differentiation were investigated. AS osteoprogenitors exposed to DHT during osteoblast differentiation showed no differences in intercellular ALP activity or ALP staining (Fig. 2a); however, calcium deposits were decreased based on ARS staining and quantification (Fig. 2b). In addition, osteoprogenitor cells in disease controls did not differ in osteogenic activities and calcium deposits after treatment with DHT (Supplementary Figure). Accordingly, the osteocalcin (*OCN*) expression was decreased, and *DKK1* and *SOST* expression was increased at the mRNA level in DHT-treated AS progenitor cells (Fig. 2c).

**Fig. 3:**
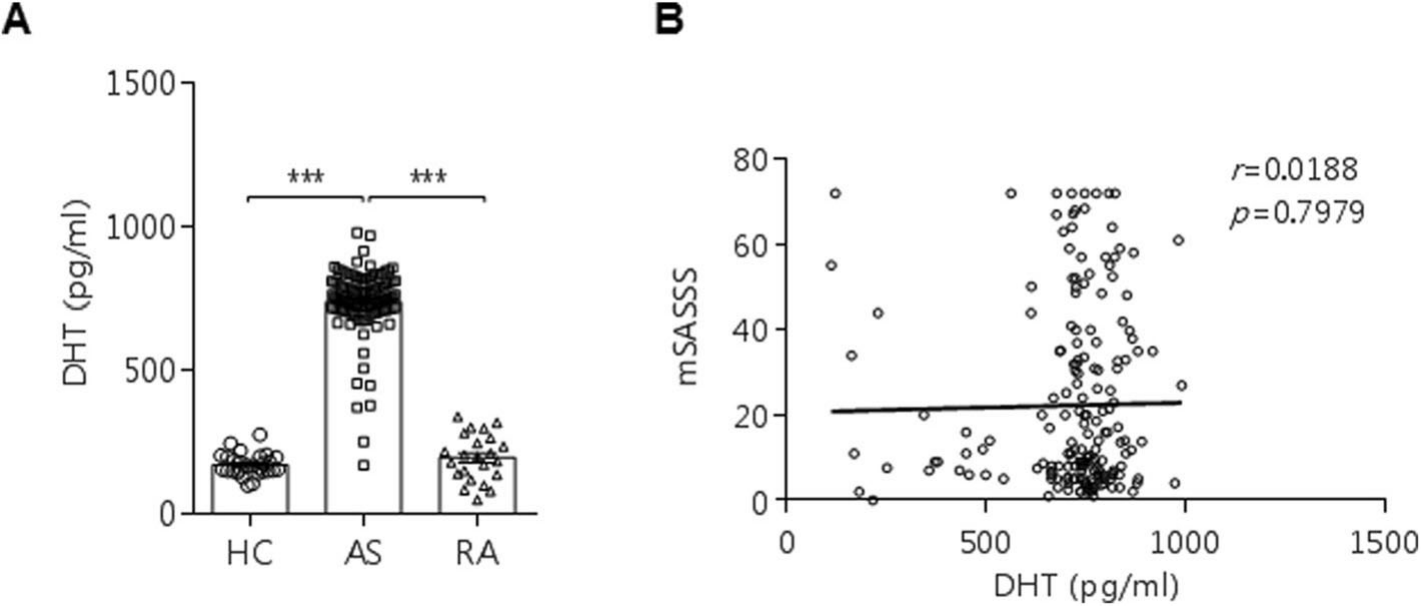
DHT level and mSASSS in patients with AS. **a** DHT levels in HC (*n* = 28), AS (*n* = 189), and RA patients (*n* = 23). **b** The lack of correlation of DHT levels with mSASSS in AS patients

DHT level in AS patients was significantly higher than that in other groups (Fig. 3a). However, DHT level was not correlated with radiographic progression measured based on mSASSS (Fig. 3b).

## Discussion

In the present study, dutasteride treatment resulted in severe spinal ankylosis in curdlan-administered SKG mice with increased population of IL-17A-secreting cells. In a recent report, IL-17A levels and T_H_17 frequency were significantly higher in male AS patients than in female AS patients [9]; however, testosterone concentration was negatively associated with activation of the T_H_17/IL-17 axis [23, 24]. If testosterone is increased to compensate for the activity of the T_H_17/IL-17 axis, suppression of testosterone might cause unexpected effects on the activation of the T_H_17/IL-17 axis. In the present study, an active metabolite converted from testosterone, DHT, was iatrogenically suppressed by dutasteride resulting in increased spinal mineralization in curdlan-administered SKG mice, mimicking spondyloarthritis. The intensity of mineralization was positively correlated with serum IL-17A levels, and dutasteride increased the number of viable IL-17A-secreting cells in curdlan-administered SKG mice, indicating dutasteride treatment in the in vivo model may exacerbate the pathogenic features of AS characterized by increased IL-17A and accumulation of spinal mineralization.

Clinically, anti-IL-17A antibodies are more effective for controlling the radiographic progression of AS compared with anti-TNF antibodies over a 2-year period [25], and evidence indicates IL-17A induces osteoblast differentiation from progenitor cells of AS patients [10]. The SKG mouse model is dependent on T_H_17/IL-17-inducible axial spondyloarthritis [12]. In the present study, dutasteride increased the proportion of viable IL-17A-secreting cells in a mouse model; however, the population of T_H_17 cells was not increased in the dutasteride group compared with the curdlan group. Because the cellular sources of IL-17A are lymphocytes, including CD4^+^, CD8^+^, γδ-T, invariant NKT, innate lymphoid cells, and non-T cells including neutrophils [26], the increased levels of IL-17A secretory cells caused by dutasteride might not be dependent on T_H_17 cells. However, whether the increased population of these cells resulted only from the effects of dutasteride could not be determined. Regarding osteoblasts, deleterious effects of anti-IL-17A on bone mass could be a concern although the effects of IL-17A on osteoporosis in the SKG mouse model were unclear. However, IL-17A is also a potent activator of osteoclast differentiation [27, 28], which triggers osteoporosis.

Primary bone-derived cells from the facet joints of AS patients were used to examine the effects of DHT and dutasteride in vitro. Dutasteride treatment did not show any significant changes in AS osteoprogenitor cells under osteogenic differentiation conditions (data not shown). However, a significant reduction of calcified nodules was observed under osteogenic conditions stimulated with DHT, although ALP activity was not different, as shown in Fig. 2. DHT treatment for 7 days under osteogenic conditions markedly increased the mRNA expression of *DKK1* and *SOST* and decreased *OCN* expression. Therefore, DHT treatment in AS osteoprogenitor cells delayed the physiological changes of osteogenic differentiation by activating *DKK1* and *SOST* expression. DHT was higher in the serum of AS patients than in RA patients or healthy controls; however, cross-sectional DHT levels were not correlated with mSASSS (Fig. 3b). In agreement with the results from the present study, the testosterone level did not correlate with mSASSS scores in male AS patients [29]. Therefore, the association of male hormone levels with mSASSS should be investigated in a serial follow-up or a large retrospective study.

The role of DKK1 or SOST in bone biology as potent antagonists of the Wnt signaling pathway has been previously reported [30]. Specifically, secreted DKK1 and SOST negatively regulate the LRP5/6 receptors of osteoblasts and inhibit Wnt-mediated bone formation by acting at several differentiation stages of the osteoblasts. Transgenic *SOST*-knockout mice display a high bone mass with increased bone mineralization [31, 32]. In several clinical studies, serum SOST levels were reduced in AS patients, and SOST has been proposed as a marker of radiographic progression. Furthermore, SOST levels were increased by treatment with an anti-TNF inhibitor [33]. Taken together, these data indicate the increased bone formation in the spinal region of mice in the current study could be due to reduced serum SOST levels caused by dutasteride treatment.

Several AS patients experience hair loss, for which dutasteride could be prescribed concomitantly. Despite the clinical relevance, the effects of dutasteride on the disease progression of patients with AS have not been determined in previous studies. In the present study, the possible harmful effects of dutasteride on bone formation in AS were presented. The experimental dutasteride diet in representative male SKG mice was designed to mimic human AS disease in vivo. The increased number of IL-17A-secreting cells and IL-17A cytokine levels and decreased SOST levels in response to dutasteride are largely consistent with the pathogenic factors associated with AS.

The present study had several limitations. First, the precise mechanism by which IL-17A-secreting cells and IL-17 cytokines were increased by dutasteride could not be determined. Second, the mechanism of androgen receptor-regulation mediated by DHT in primary cells was not clearly defined; thus, further research is needed to better understand the underlying actions of regulatory male hormones. In addition, clinical studies are needed to verify the effects on the spinal structure in AS patients undergoing dutasteride therapy for prostate cancer. Third, the AS osteoprogenitors investigated in the present study might be inappropriate to explain the complete mechanism of syndesmophyte formation; however, the focus was on osteoblast proliferation, which was the reason for choosing the particular AS osteoprogenitors. Finally, an alternative mechanism of severe ankylosis caused by dutasteride, such as microbiota, was not fully evaluated. Imbalance of gut microbiota was suggested as a possible pathogenesis in a HLA-B27-induced spondyloarthritis model [34]. In addition, a sex-related distinct population of microbiota during development of inflammatory bowel diseases has recently been reported [35]. Therefore, effects of dutasteride on the imbalance of microbiota should be studied in the future.

## Conclusion

Treatment with a DHT inhibitor in curdlan-administered SKG mice led to increased mineralization of the spine in vivo, and DHT treatment attenuated osteoblast differentiation in vitro. Therefore, the prescription of regimens inhibiting DHT in AS patients warrants careful consideration regarding the progression of spinal ankylosis.

## Data Availability

All data generated and analyzed during this study are included in this article. Materials used in this study are available from the corresponding author on reasonable request.

## Abbreviations

AS: Ankylosing spondylitis
HC: Healthy control
RA: Rheumatoid arthritis
mSASSS: Modified Stoke Ankylosing Spondylitis Spinal Score
DHT: Dihydrotestosterone
ALP: Alkaline phosphatase
ARS: Alizarin red staining
IL-17A: Interleukin-17A
DKK1: Dickkopf-related protein 1
SOST: Sclerostin
OCN: Osteocalcin
OPG: Osteoprotegerin
RANKL: Receptor activator of nuclear factor kappa B ligand
